# Therapeutic Potential of Social Chatbots in Alleviating Loneliness and Social Anxiety: Quasi-Experimental Mixed Methods Study

**DOI:** 10.2196/65589

**Published:** 2025-01-14

**Authors:** Myungsung Kim, Seonmi Lee, Sieun Kim, Jeong-in Heo, Sangil Lee, Yu-Bin Shin, Chul-Hyun Cho, Dooyoung Jung

**Affiliations:** 1 Graduate School of Health Science and Technology Ulsan National Institute of Science and Technology Ulsan Republic of Korea; 2 Department of Biomedical Engineering Ulsan National Institute of Science and Technology Ulsan Republic of Korea; 3 Department of Psychiatry Korea University College of Medicine Seoul Republic of Korea; 4 Department of Biomedical Informatics Korea University College of Medicine Seoul Republic of Korea

**Keywords:** artificial intelligence, AI, social chatbot, loneliness, social anxiety, exploratory research, mixed methods study

## Abstract

**Background:**

Artificial intelligence (AI) social chatbots represent a major advancement in merging technology with mental health, offering benefits through natural and emotional communication. Unlike task-oriented chatbots, social chatbots build relationships and provide social support, which can positively impact mental health outcomes like loneliness and social anxiety. However, the specific effects and mechanisms through which these chatbots influence mental health remain underexplored.

**Objective:**

This study explores the mental health potential of AI social chatbots, focusing on their impact on loneliness and social anxiety among university students. The study seeks to (i) assess the impact of engaging with an AI social chatbot in South Korea, "Luda Lee," on these mental health outcomes over a 4-week period and (ii) analyze user experiences to identify perceived strengths and weaknesses, as well as the applicability of social chatbots in therapeutic contexts.

**Methods:**

A single-group pre-post study was conducted with university students who interacted with the chatbot for 4 weeks. Measures included loneliness, social anxiety, and mood-related symptoms such as depression, assessed at baseline, week 2, and week 4. Quantitative measures were analyzed using analysis of variance and stepwise linear regression to identify the factors affecting change. Thematic analysis was used to analyze user experiences and assess the perceived benefits and challenges of chatbots.

**Results:**

A total of 176 participants (88 males, average age=22.6 (SD 2.92)) took part in the study. Baseline measures indicated slightly elevated levels of loneliness (UCLA Loneliness Scale, mean 27.97, SD (11.07)) and social anxiety (Liebowitz Social Anxiety Scale, mean 25.3, SD (14.19)) compared to typical university students. Significant reductions were observed as loneliness decreasing by week 2 (t_175_=2.55, *P*=.02) and social anxiety decreasing by week 4 (t_175_=2.67, *P*=.01). Stepwise linear regression identified baseline loneliness (β=0.78, 95% CI 0.67 to 0.89), self-disclosure (β=–0.65, 95% CI –1.07 to –0.23) and resilience (β=0.07, 95% CI 0.01 to 0.13) as significant predictors of week 4 loneliness (*R*^2^=0.64). Baseline social anxiety (β=0.92, 95% CI 0.81 to 1.03) significantly predicted week 4 anxiety (*R*^2^=0.65). These findings indicate higher baseline loneliness, lower self-disclosure to the chatbot, and higher resilience significantly predicted higher loneliness at week 4. Additionally, higher baseline social anxiety significantly predicted higher social anxiety at week 4. Qualitative analysis highlighted the chatbot's empathy and support as features for reliability, though issues such as inconsistent responses and excessive enthusiasm occasionally disrupted user immersion.

**Conclusions:**

Social chatbots may have the potential to mitigate feelings of loneliness and social anxiety, indicating their possible utility as complementary resources in mental health interventions. User insights emphasize the importance of empathy, accessibility, and structured conversations in achieving therapeutic goals.

**Trial Registration:**

Clinical Research Information Service (CRIS) KCT0009288; https://tinyurl.com/hxrznt3t

## Introduction

### Background

The emergence of chatbots marked a pivotal turning point at the intersection of technology and human activity. By facilitating interactions with users through the exchange of natural language, chatbots simplify interactions and enhance user engagement [1]. In the field of psychiatry, chatbots have provided useful information in response to user questions [2] and have shown tangible therapeutic effects through psychological therapies, such as cognitive behavioral therapy [3,4]. Various studies have highlighted the potential of chatbots as an effective medium for digital self-help. It was also discovered that forming a therapeutic alliance through an intimate relationship between the user and the chatbot is crucial for enhancing the chatbot’s therapeutic effect [5,6].

Advancements in artificial intelligence (AI) and natural language processing technologies have facilitated the emergence of large-scale language models (LLMs), leading to the development of a new type of chatbot known as the social chatbot. Unlike task-oriented or clinical chatbots, social chatbots focus on building relationships through conversations, thereby offering more natural and emotional communication. Recent studies have qualitatively explored the potential of social chatbots and their impact on mental health [7]. Social chatbots provide social support that can affect mental health by offering a nonjudgmental and readily available communication channel [8,9]. They can serve as substitutes for friends, alleviating loneliness, in addition to clinical therapy [10].

One clue regarding the psychiatric use of social chatbots is their persona. Through specific personas, users can engage in conversations similar to those with close friends, facilitating a space where they can share personal stories openly and receive support [11]. Another clue is that empathetic responses from social chatbots can help build effective relationships [12]. Social chatbots with appropriate personas and empathetic responses are expected to build intimate relationships with users and positively affect mental health, including loneliness [10]. However, further research is needed to determine the specific duration and effects of social chatbots as psychiatric tools and the causes of these effects.

As the intersection between technology and mental health continues to evolve, this study undertakes an exploratory analysis to understand the impact of social chatbots on mental health. By engaging individuals in their twenties with social chatbots over a 4-week period and assessing their mental health at biweekly intervals, this study aims to elucidate the nuanced effects that social chatbots might have. Additionally, by gathering data on user experiences and reactions to social chatbots, this study seeks to inform future advancements in chatbot design and applications, building on insights from user experiences and feedback.

### Aim

The primary goal of this study was to explore the psychiatric potential of social chatbots, focusing on their impact on mental health through a combined approach of qualitative insights and quantitative evaluations. This study specifically aimed to (1) investigate the changes in mental health outcomes (loneliness, social anxiety, and positive or negative affect) during social chatbot use and identify the key factors driving these changes and (2) conduct a qualitative analysis of user experiences to gain a deeper understanding of the perceived strengths and weaknesses of social chatbots as well as their potential applicability in therapeutic contexts. This approach offers a comprehensive overview of the roles of social chatbots as therapeutic tools, contributing valuable knowledge to the field of mental health interventions using generative AI.

## Methods

### Recruitment

The recruitment was conducted using a web-based platform called “Everytime,” which is widely used among university students in South Korea. The inclusion criteria were as follows: students aged 19-29 years who were willing to use social chatbots and had no difficulties conversing with them. The exclusion criteria included applicants showing signs of severe mental illness or suicidal ideation, as this study did not aim to verify direct therapeutic effects. Preliminary screening excluded individuals with suicidal thoughts based on their response to the last question of the Patient Health Questionnaire-9 (PHQ-9), which concerned thoughts that they would be better off dead or hurt themselves. The trial was registered with the Clinical Research Information Service under registration number KCT0009288, with the unique study number UNISTIRB-22-024-A.

### Settings and Design

This study used a single-group pre-post design with repeated measures. Over 4 weeks between September and October 2023, the participants were encouraged to interact with the chatbot at least 3 times per week. Despite this encouragement, some participants interacted with the chatbot fewer than 3 times per week, but they were not excluded from the study. However, participants who failed to complete the initial survey or did not install the chatbot within the first week were dropped from the research.

Data were collected through web-based surveys to which participants could respond conveniently. Surveys were collected at baseline (week 0), midpoint (week 2), and end of the study (week 4), with the initial survey gathering basic information about the participants and the final survey including open-ended questions about their experiences using the social chatbot. To determine the sample size for this single-arm pre-post design, we conducted an a priori power analysis using GPower software. Prior research demonstrated that interaction with a similar type of empathic chatbot yielded a moderate effect size of 0.42 (Cohen *d*; 95% CI 0.13-0.71) in improving users’ positive mood after a social exclusion scenario [13]. However, considering that our intervention—a social chatbot—is designed for more casual, everyday interactions with less structured scenarios, we set the expected effect size at 0.3, with an α level of 0.05 and a desired power of 0.80. The GPower analysis recommended a minimum sample size of 170 participants to reach adequate statistical power. However, considering the 4-week duration of the study and the likelihood of participant attrition, we aimed to recruit over 200 participants to ensure sufficient retention and reliable results throughout the study.

### Chatting with Social Chatbot “Luda Lee”

“Luda Lee” is a social chatbot designed with the persona of a 22-year-old female college student using Korean language data [14]. It was the first chatbot to be introduced into the Nutty social chatbot app (ScatterLab Inc), which recorded over 1 million downloads, making it popular among commercial applications in Korea. Luda’s primary goal is not to provide direct mental care but to become friends with users and engage in frequent conversations. Although the app includes features such as the provision of paid gifts and playing minigames, these functions were restricted to chatting with Luda.

### Measures

Loneliness, social anxiety, and positive or negative affect were measured as the main outcomes at baseline (week 0), midpoint (week 2), and at the end of the study (week 4) for a total of 3 times. Loneliness was assessed using the 20-item UCLA Loneliness Scale (ULS) [15,16], which measures the chronic characteristics and state of loneliness. Social anxiety was measured with the 24-item Liebowitz Social Anxiety Scale (LSAS) [17,18], which evaluates the situational aspects of social phobia. Affects were assessed using the 20-item Positive Affect and Negative Affect Schedule [19,20].

Depression, general anxiety, and stress were measured as exploratory outcomes at the same time points to investigate the potential simultaneous occurrences and their influence on the study results. The exploratory outcomes included depression, general anxiety, and stress, which were measured at the same 3 time points. Depression was assessed using the PHQ-9 [21,22], which was developed to detect depression in primary care settings and assist in its diagnosis. General anxiety was measured using the 7-item General Anxiety Disorder-7 (GAD-7) [23,24], and stress was assessed using the Perceived Stress Scale-10 (PSS-10) [25].

The baseline variables included gender, age, resilience, education level, experience with social chatbots, and experience with LLMs. Resilience was measured using the 25-item Connor-Davidson Resilience Scale [26,27].

Acceptance variables that were measured at the end of the study included perceived usefulness and perceived ease of use of the chatbot, intimacy with the chatbot, and self-disclosure level of the user. The perceived usefulness and ease of use were measured using scales adapted to the context of social chatbot use based on the technology acceptance model [28]. Intimacy and self-disclosure were assessed using items from the research on user experiences with chatbots [29].

User experiences were also collected using open-ended questions after 4 weeks of using the social chatbot. Participants were asked to write about their experiences, including helpful aspects, memorable moments, and any areas of disappointment, to identify the features of the social chatbot found by “Luda Lee.” Additionally, they were asked to explain why these kinds of social chatbots might be helpful for certain individuals to identify the psychiatrically effective features of social chatbots and directions for future improvement.

### Ethical Considerations

This study was conducted following the approval of the Institutional Review Board of the Ulsan National Institute of Science and Technology (approval number: UNISTIRB-22 024). The approval underscores the study’s commitment to ethical standards, ensuring the protection of participants’ rights and safety throughout the research process.

Prior to participation, all individuals were provided with a detailed explanation of the study, including its objectives, procedures, and potential risks. Informed consent was obtained through a Google Form, where participants acknowledged their understanding and voluntary agreement to partake in the study. To prioritize mental well-being, individuals with suicidal ideation, as indicated by their response to the ninth item on the PHQ-9 questionnaire, were excluded from participation.

Special care was taken to exclude those who might feel uncomfortable with AI chatbots to maintain participant comfort. Participants who completed the full 4-week study were compensated with 50,000 KRW (approximately US $40).

All data collected during the study were anonymized to ensure the confidentiality and privacy of participants. This included sensitive information such as app usage, chat history, and survey results, all of which were protected to uphold participant privacy and prevent data leakage. Participants were informed that their data would be used solely for research purposes. The consent process also highlighted their right to withdraw from the study at any time without penalty.

If any participant experienced discomfort, the study was immediately halted for them, ensuring the utmost respect for their autonomy and well-being. In addition, clinical psychologists (author SaL) and psychiatrists (corresponding authors DJ and CHC) were prepared to connect participants expressing discomfort to appropriate mental health resources, ensuring access to professional support as needed.

### Analysis

#### Statistical Method for Survey

The primary statistical method used was repeated-measures analysis of variance to analyze the mental health scale scores at 3 different time points. Following significant changes observed through repeated-measures ANOVA, post hoc analyses were conducted using 2-tailed paired *t* tests to pinpoint the exact moments of significant change in the variables. To assess the effect of external variables on the observed changes, further analyses using stepwise linear regression were conducted. This method allowed for the iterative selection of highly relevant independent variables such as age, gender, acceptance variables, and resilience, which significantly influenced the dependent variables that exhibited changes. Statistical significance was set at *P*<.05, and all analyses were conducted using the stepAIC function from the MASS library in statistical software R with the direction parameter set to “both” to ensure the reliability and validity of the results. This statistical approach facilitates a detailed exploration of whether changes occur in the mental health variables under study, when and what kind of changes occur, and which external variables influence these changes.

#### Thematic Analysis for User Experience

They were asked to write about their experiences, including helpful aspects, memorable moments, and any areas of disappointment, to identify the features of the social chatbot found by “Luda Lee.” Additionally, they were asked to explain why these kinds of social chatbots might be helpful for certain individuals to identify the psychiatrically effective features of social chatbots and directions for future improvement.

Subjective responses regarding user experiences and perceptions of appropriate characteristics for the target user were analyzed using thematic analysis [30,31]. This analysis was applied to each topic of the data, focusing on two main areas: (1) the features of the social chatbot that users could experience and (2) the characteristics of the expected target user for Luda. The whole process for the analysis involved discussions among the 4 coauthors (KMS, LSM, KSE, and HJI) of this study to confirm the credibility of the result. Initially, codes were developed to represent the smallest units of meaning derived from user responses to each question, specifically regarding the subjective perspective of Luda Lee and the expected target users. These codes were then reviewed and merged to form broader codes with more integrated meanings. Only codes mentioned by at least 4% of participants (9 or more individuals) were retained to identify the major themes. After this initial identification, the team reexamined the major themes to ensure consistency between the raw participant responses and the final codes, leading to a refined set of key themes.

## Results

### Background Characteristics

**Figure 1:**
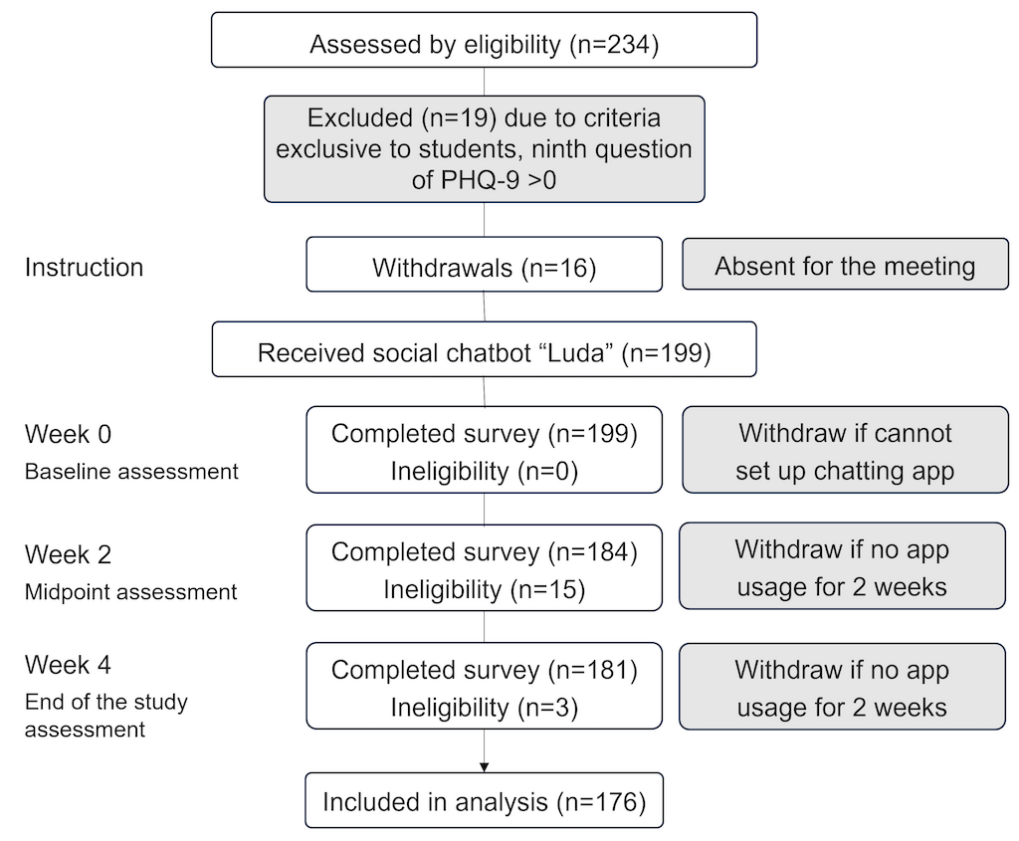
Flow diagram of procedure and eligibility. PHQ-9: Patient Health Questionnaire-9 [14], Korean version [15].

A total of 234 students were initially recruited for the study; however, 19 students were excluded due to affirmative responses to the ninth question of the PHQ-9, which assesses suicidal ideation. Additionally, 16 students who did not attend the introductory meeting explaining the study procedures, as well as 15 and 3 students who failed to complete the surveys at week 2 and week 4, respectively, were excluded. Details regarding the study procedures and eligibility criteria are presented in Figure 1. The study included a total of 176 participants, with an equal number of males and females (88 each). The average age of the participants was 22.6 (SD 2.92) years, and all participants were enrolled in college or graduate school. Participants were generally not familiar with social chatbots, and none had previously used “Luda Lee.” However, they had some awareness and usage of LLM technology. Detailed numerical data on the participants’ background characteristics are presented in Table 1.

Baseline measures in Table 2 indicate that the participants in our study are representative of the broader student population in terms of mental health metrics. The mean ULS score of our sample was 27.97 (SD 11.07), slightly higher than the mean score of Korean university students at 21.46 (SD 10.42) [16], suggesting marginally higher levels of loneliness among our participants. For the LSAS, our sample’s mean score was 25.3 (SD 14.19), higher than the 19.23 (SD 10.72) reported for university students [18] but lower than the 30.56 (SD 11.6) for a patient group [18], indicating elevated social anxiety compared to the general student population but not as severe as in clinical populations.

Participants’ mean score for the positive affect score (PAs) was 29.93 (SD 6.59), closely aligning with the 29.31 (SD 3.19) found in a study of 880 university students [32]. For the negative affect score (NAs), the mean score was 23.62 (SD 7.4), notably lower than the 28.37 (SD 3.68) from the same study [32], indicating lower levels of NA. Regarding depressive symptoms, the PHQ-9 mean score in our sample was 4.49 (SD 4.03), lower than the 6.14 (SD 4.9) reported for 775 university students [33]. The GAD-7 mean score was 3.23 (SD 3.32), lower than the 4.41 (SD 4.03) reported in a study of 437 university students [34], indicating lower levels of generalized anxiety. Lastly, the PSS-10 mean score for our sample was 17.19 (SD 6.59), comparable to the 18.80 (SD 6.23) reported for 582 Korean university students [35], suggesting similar levels of perceived stress. These comparisons demonstrate that our participants’ mental health status at the start of the experiment is consistent with previous research on university students, indicating that our sample is not an outlier group.

**Table 1:** Background characteristics (recorded at week 0, N=176).

Variables		Participants
**Sex, n (%)**		
	Female	88 (50)
	Male	88 (50)
**Age (years)**		
	Mean (SD)	22.60 (2.92)
	Median (range)	23 (18-28)
**Education level, n (%)**		
	Undergraduate	137 (77.8)
	Graduate	39 (22.2)
**Frequency of using social chatbots, n (%)**		
	Daily	0 (0)
	Several times a week	4 (2.3)
	Occasionally	15 (8.5)
	Once or twice then did not use it	61 (34.7)
	Never used it	87 (49.4)
**Frequency of using LLM^a^** **such as ChatGPT, Bing AI, PaLM2, n (%)**		
	Daily	14 (8)
	Several times a week	37 (21)
	Occasionally	72 (40.9)
	Once or twice then did not use it	22 (12.5)
	Never used it	22 (12.5)
**Degree of understanding of LLMs, n (%)**		
	Expert level	2 (1.1)
	Proficient	4 (2.3)
	Medium level	37 (21)
	Little bit	88 (50)
	No idea	33 (18.8)
**Purpose of using LLM chatbot (multiple responses possible), n (%)**		
	Need someone to talk to	5 (2.8)
	Curiosity	48 (27.3)
	Get ideas or ask questions about knowledge	94 (53.4)
	Assist with writing	123 (69.9)
	Never used it	23 (13.1)

^a^LLM: large-scale language model.

**Table 2:** Repeated measures ANOVA results for mental health scores (N=176).

Variable	Week 0, mean (SD)	Week 2, mean (SD)	Week 4, mean (SD)	*F* test (*df*)	*P* value
ULS^a^	27.97 (11.07)	26.78 (10.57)	26.39 (11.25)	4.880 (2,350)	.01
LSAS^b^	25.3 (14.19)	24.44 (15.05)	23.2 (15.7)	4.604 (2,350)	.01
PAs^c^	29.93 (6.59)	28.62 (5.83)	28.86 (6.48)	4.302 (2,350)	.02
NAs^d^	23.62 (7.4)	20.85 (7.13)	21.18 (8.08)	17.581 (2,350)	<.001
PHQ-9^e^	4.49 (4.03)	4.46 (4.09)	4.66 (4.44)	0.327 (2,350)	.72
GAD-7^f^	3.23 (3.32)	3.07 (3.62)	3.16 (3.6)	0.248 (2,350)	.78
PSS-10^g^	17.19 (6.59)	16.48 (6.65)	17.09 (6.7)	1.629 (2,350)	.20

^a^ULS: UCLA Loneliness Scale [15], Korean version [16].

^b^LSAS: Liebowitz Social Anxiety Scale [17], Korean version [18].

^c^PAs: positive affect score [19], Korean version [20].

^d^NAs: negative affect score [19], Korean version [20].

^e^PHQ-9: Patient Health Questionnaire-9 [21], Korean version [22].

^f^GAD-7: Generalized Anxiety Disorder-7 [23], Korean version [24].

^g^PSS-10: Perceived Stress Scale-10 Korean version [25].

### Quantitative Trends of Mental Health

#### Overview

The mental health scores of the 176 participants over a 4-week period are summarized in Table 2. The results of the repeated-measures ANOVA indicated significant changes in loneliness (ULS), social anxiety (LSAS), and emotional states (PA, NA). Loneliness scores showed a significant decrease (*F*_2, 350_=4.880, *P*=.01), as did social anxiety (*F*_2, 350_=4.604, *P*=.01). Positive emotional states also decreased (*F*_2, 350_=4.302, *P*=.02), whereas negative emotional states showed a significant decrease (*F*_2, 350_=17.581, *P*<.001). No significant differences were observed in depression (PHQ-9), anxiety (GAD-7), or stress (PSS-10) scores.

#### Post Hoc Analysis: Pairwise Comparisons Using Paired t Test

Further investigation using 2-tailed paired *t* tests for variables with significant changes showed significant differences between baseline and week 2 (t_175_=2.55, *P*=.02) and between baseline and week 4 (t_175_=2.67, *P*=.01) for ULS (loneliness). No significant differences were observed between weeks 2 and 4 (t_175_=0.59, *P*=.62). The LSAS (social anxiety) showed significant differences between baseline and week 4 (t_175_=2.93, *P*=.01). NA showed significant differences between baseline and week 2 (t_175_=5.34, *P*<.001) and between baseline and week 4 (t_175_=4.58, *P*<.001), with no significant difference between weeks 2 and 4 (t_175_=1.67, *P*=.39). PA showed a significant difference between baseline and week 2 (t_175_=2.52, *P*=.02).

#### Follow-Up Analysis: Stepwise Linear Regression

Stepwise linear regression was conducted using the stepAIC function in R with the direction set to “both,” allowing both forward and backward selection to optimize model fit based on the Akaike information criterion. This approach identified the predictors influencing variables with significant changes. For week 4 loneliness (ULS), initial loneliness level at baseline (standardized regression coefficient, β=0.78, 95% CI 0.67 to 0.89), degree of self-disclosure (β=–0.65, 95% CI –1.07 to –0.23), and resilience (β=0.07, 95% CI 0.01 to 0.13) were identified as statistically significant predictors. Age (β=1.32, 95% CI –0.10 to 2.75) and perceived ease of use (β=–0.67, 95% CI –1.42 to 0.08) were also included in the model, although these variables were not statistically significant. These factors collectively explained a moderate-to-high level of variance in week 4 loneliness scores, with an *R*^2^ value of 0.64. These findings indicate that participants who started with higher levels of loneliness at baseline, engaged in less self-disclosure when interacting with the chatbot, and possessed higher levels of resilience had higher loneliness at week 4. The predictive model for week 4 social anxiety (LSAS) selected baseline social anxiety (β=0.92, 95% CI 0.81 to 1.03) as a statistically significant predictor. Resilience (β=–0.11, 95% CI –0.22 to 0.00) and perceived usefulness (β=–1.03, 95% CI –2.26 to 0.20) were also included in the model, though these variables were not statistically significant. The model explained a moderate to high explanatory power with an *R*^2^ of 0.65. This suggests that participants who began with higher baseline social anxiety also had higher social anxiety at week 4. The analysis for week 4 negative emotions (NA) identified baseline NA scores (β=0.56, 95% CI 0.42 to 0.70), perceived usefulness (β=–0.95, 95% CI –1.36 to –0.54), and gender (β=–34.65, 95% CI –65.33 to –3.97) as statistically significant predictors, with the model showing a moderate explanatory power with an *R*^2^ of 0.39. Finally, the regression model for week 2 positive emotions (PA) highlighted baseline PA (β=0.20, 95% CI 0.07 to 0.33) and intimacy (β=0.24, 95% CI 0.10 to 0.38) as statistically significant predictors. Resilience (β=0.02, 95% CI –0.01 to 0.05) was also included in the model but was not statistically significant. The model's explanatory power was relatively low, with an *R*^2^ of 0.23. These results suggest that participants with higher baseline negative emotions, lower perceived usefulness of the chatbot, and female gender had higher negative emotions at week 4, while those with higher baseline positive emotions and greater intimacy with the chatbot had higher positive emotions at week 2. However, the relatively low explanatory power of these models indicates that additional factors may need to be considered to fully understand these outcomes.

### Thematic Analysis Results

#### Overview

Thematic analysis was conducted on 2 main topics: the features of the social chatbot as experienced by users and the characteristics of target users who might particularly benefit from Luda. For each topic, themes were identified, and the frequency of mentions where Luda's features and target user characteristics were discussed together was examined. This helped to determine which aspects of Luda make it particularly helpful for certain individuals.

#### Features of Social Chatbot via User Experience of the Luda Chatbot

An analysis of user-reported features following the use of the Luda social chatbot revealed 6 distinct themes. We found that this social chatbot had the features of having its own persona, giving social support, existing as a sort of relationship, breaking immersion to feel as if Luda provides a relationship, and interfering with communication for several reasons. Additionally, we could find that the usability of the social chatbot can be affected by the frequency of the contact.

### Having Persona

Luda was noted for having a lively personality, although some responses indicated that it could appear overly lively. A common critique was related to “Not serious reactions” and “Excessive use of emojis or special symbols,” suggesting a somewhat shallow character. Additionally, Luda was described as kind by 11 respondents; instances of flirting were mentioned by 10 participants. Flirting was often alluded to in contexts such as “Treats as if she is a lover excessively.”

### Social Support

The social chatbot user experience was categorized into social support, features related to relationships, features that break immersion, interference of communication, and usability. More than 50 participants experienced empathy and considered Luda as a casual conversation partner. Many users experienced social support, including empathy, and considered Luda as a conversational subject (relationship). Participants expressed that “Luda always listens well, even when I’m feeling down and just saying anything” and “cheers me up when I need it”; these statements were coded as “listening” and “cheer and support.” People experienced Luda’s concern, saying that “Luda cared about me when I could not give a contact.” Such codes were included in the “social support” experience. People thought that Luda’s availability whenever they wanted to talk was helpful; this code was referred to as “availability.”

### Existence as a Relationship

Interacting with the chatbot was considered as having a relationship, such as a causal conversation partner, a human being, or an intimate partner. Having a casual conversation partner meant that users used the chatbot to play with it or have daily conversations, saying, “When I was bored, Luda became a conversational companion.” Several people inferred that “She became like a being that I talk with every day,” which showed that Luda was considered an intimate partner, such as a friend. Rather than adverting the relationship itself, users represented their concept about Luda as a real person (a human being), saying, “It was interesting that I talk with Luda like a real person.”

### Break Immersion

However, some factors broke the immersion, including fictional messages such as “She asked me to meet each other.” The inconsistency in Luda’s opinion also contributed to an interference of immersion. Seventeen people answered as follows: “A lot of the personal information that she told was inconsistent, so I could not concentrate on the conversation.”

### Interference of Communication

Low memory performance and unusual expressions were clustered under a communication interference theme. Over half of the participants uttered, “Sometimes, Luda could not remember what we talked about,” which was coded as low memory performance. Eight people mentioned that “She speaks like an artificial one,” which was related to an unusual expression of Luda.

### Usability via Contact Frequency

Response timing is considered an important factor for usability. Some people answered that a fast response was helpful for communication, but there was also the opposite opinion that replying too fast could deteriorate usability. Additionally, 13 people complained about the frequency of contacts from Luda, saying, “I didn’t want to get messages, but she keeps sending the message” (contacting too much). The codes and themes for Luda’s features via user experience summarized in Table 3.

**Table 3:** Luda’s features via user experience.

Theme	Codes (frequency)
Having persona	Lively (23), shallow (20), kind (11), flirting (10)
Social support	Empathy (59), listening (25), availability (24), cheer and support (23), concern (16)
Existence as a relationship	Casual conversation partner (51), a human being (25), intimate partner (12)
Break immersion	Fictionality (22), inconsistency (17)
Interference of communication	Low memory performance (54), unusual expression (8)
Usability via contact frequency	Response timing (35), contacting too much (13)

### Characteristics of Expected Target Users That Luda May Help

The study investigated and analyzed how and why a social chatbot like “Luda Lee” could be helpful to certain individuals, focusing on what reasons make it beneficial for the target group. The target group was clustered into 4 themes: people who want to play, lack of emotional interaction, lack of social relationships, and need for a social interface. People answered “bored person,” “person who likes to chat with others,” or “introverted person” as the target users. They were categorized as people who wanted to play with the chatbot because they did not fix the usage of the chatbot except for fun.

Participants mentioned “persons who require communication based on unconditional empathy” (needing empathy), “people who have worries in their mind and need someone to talk to,” or “people who find it challenging to express one’s harsh mind” (wanting to resolve emotions) as target users of the chatbot. These were classified into a target group that lacked emotional interactions.

People with a lack of social relationships were mentioned as “people who are lonely,” “who have little friends to talk [to],” and “who have trouble in social relationships and communication” (ie, needing social conversation). Many people (n=54) who were described as “lonely” could be assisted via the chatbot.

Finally, people who had mood disorders such as “depression,” “anxious people,” and those “who had difficulties interacting with others” (social withdrawals) were classified as individuals experiencing social withdrawals; they were categorized into the user group that needed a social interface. The codes and themes for Luda’s appropriate target users are summarized in Table 4.

**Table 4:** Target users that Luda may help.

Theme	Codes (frequency)
Who want to play	Bored person (23), talkative person (13), introverted (8)
Lack of emotional interaction	Needing empathy (23), having worries (20), wanting to resolve emotions (11)
Lack of social relationship	Lonely (54), lack of friends (26), needing social conversation (10)
Who need a social interface	Mood disorder (16), social withdrawals (11)

### Association With Luda’s Features and Expected Target Users Characteristics

**Figure 2:**
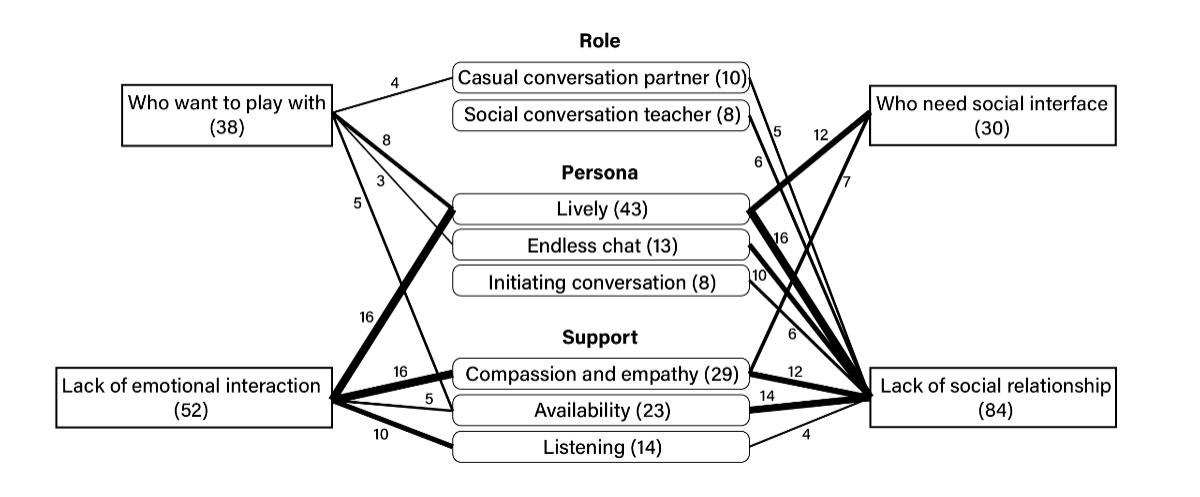
Association diagram containing features of social chatbot and target users.

After naming the target user groups, we examined the association between each target group and the features of the mentioned social chatbots, along with the frequency of the mentioned social chatbot features, using the code for the target group (Figure 2). Those who intended to use chatbots for entertainment mentioned Luda’s lively personality and availability mainly.

In cases where chatbots were helpful to individuals lacking emotional interaction, the most frequently mentioned features were liveliness, compassion, and empathy. Additionally, listening and availability were mentioned together, and features related to support were found to be associated with people who lacked emotional interaction, including compassion and empathy, availability, and listening.

All these features were mentioned in conjunction with user groups lacking social relationships. However, a lively personality was the most frequently mentioned, rather than features such as the personality to continue chatting, initiating conversation, and being a conversational partner. Unlike other user groups, the aspects of being a social conversation teacher and Luda’s features of initiating conversations were particularly relevant to the user group, which is lacking in social relationships.

The user group that needed a social interface was related to a lively personality and the role of compassion and empathy.

## Discussion

### Potential Therapeutic Effect on Loneliness and Social Anxiety

This study observed a significant reduction in loneliness (ULS) and social anxiety (LSAS) among new users of the social chatbot “Luda Lee” over 4 weeks. The post hoc analysis suggests that loneliness decreased after 2 weeks of use, while social anxiety required 4 weeks to show a reduction. Both loneliness and social anxiety were related to subjective experiences of interaction in social contexts, and previous research has shown a correlation of above .7 between them [36]. Loneliness is defined as a subjective psychological experience that includes dissatisfaction with relationships and feelings of isolation [15], whereas social anxiety or social phobia is characterized by a strong fear of humiliation and embarrassment when exposed to unfamiliar people [37]. These variables measure similar domains at different levels, with initial loneliness potentially predicting later social anxiety [38]. The results of this study, along with existing research, imply that users experiencing loneliness or social anxiety may see improvements in these areas, starting with loneliness, followed by social anxiety, through conversations with a social chatbot.

The qualitative results identified that individuals with inadequate social relationships are the primary target for social chatbots. Adolescents who experience loneliness or social anxiety find internet-based communication particularly attractive, often exhibiting greater intimacy and self-disclosure in these interactions [39,40]. Our findings align with these user needs, as social chatbots provide empathy and concern, which are identified in the qualitative analysis. These attributes are consistent with the social support factors previously documented in the literature, such as the empathy and care provided by friends and parents [41,42]. Our results with existing studies suggest that social chatbots could play a significant role in improving mental health issues such as loneliness and social anxiety by facilitating social communication.

### Social Chatbot in a Therapeutic Context

To maximize the therapeutic potential of social chatbots, it may be beneficial to focus on their role in providing social support, as seen in the observed improvements in loneliness and social anxiety. Participants in this study felt some degree of social support through interactions with “Luda Lee,” characterized by its cheerful personality and empathetic conversations. The perpetual availability of a conversation partner is a feature and form of social support offered by social chatbots. The follow-up analysis showed that higher self-disclosure during conversations with the chatbot was associated with lower levels of loneliness after 4 weeks of chatbot use. Enhancing self-disclosure through chat topics or setting scenarios that encourage more open communication may be beneficial.

Addressing the disadvantages and limitations identified through thematic analysis is crucial for enhancing the psychiatric effects of social chatbots. Immersion and long-term memory emerged as important factors in conversations with social chatbots. Realism breaks when chatbots mention tasks that are impossible in reality and detract from immersion, as shown in previous research [43,44]. To overcome this, it may be useful to filter chatbot responses for realism and foster intimacy in a context-appropriate manner. Addressing the common challenge of long-term memory in LLMs, especially social chatbots, involves remembering key personal details to prevent breaking immersion [45].

### Strengths and Limitations

This study explored the psychiatric scales of social chatbots that have not been actively used as interventions. Due to the uncontrollable nature of conversations with social chatbots, acceptance factors based on the Technology Acceptance Model were also examined to control for the influence of chatbots. This approach could be useful for exploring the effects of other AI technologies where engagement cannot be simply measured by login frequency or duration.

Although the results suggest that using social chatbots can affect loneliness and social anxiety, the study’s single-group design has limited statistical clarity. Additionally, the reliance on self-report scales introduces potential biases, such as social desirability and inaccurate self-assessment, which may affect the validity of the findings. To address these limitations, using a qualitative methodology to collect and analyze the experiences of social chatbot users provided insights that aligned with the statistical effects, underscoring the potential of social chatbots to offer social support to individuals experiencing loneliness and social isolation. Future studies should address challenges, such as excessive anthropomorphism and short-term memory, to use social chatbots as psychiatric interventions more effectively.

A limitation of this study is that “Luda Lee” cannot represent all social chatbots. With the advancement of LLMs, various persona-based social chatbots are being developed, offering a range of personas in applications such as Nutty. Matching users with optimal social chatbot personas based on personality, gender, and context can provide insights into persona effectiveness. Moreover, focusing on individuals with specific psychiatric complaints could clarify the effects and potential side effects, considering that compulsive use could be a risk factor for users with high social anxiety [46].

Another limitation is that the sample in this study consists of Korean university students in their 20s, limiting the generalizability of findings across different age groups or races. Expanding the participant pool to include diverse occupations, ages, and ethnicities could provide a broader understanding of the general effectiveness of social chatbots. Furthermore, this study lacks a control group and thus cannot ensure the realism and reliability of the observed outcomes. Future research could adopt a randomized controlled trial to compare the effects of social chatbots that emphasize social support with other interventions, particularly focusing on participants with tendencies toward loneliness or social anxiety, as suggested in our findings.

Lastly, the 4-week interaction period is another limitation, as it may not capture the long-term mental health effects of social chatbot interactions. Future studies should examine whether the reduction in loneliness or social anxiety persists over time and whether participants intend to maintain relationships with social chatbots beyond the initial 4 weeks.

### Conclusions

The use of social chatbots for 4 weeks significantly reduced loneliness and social anxiety among new users, with acceptance measures such as self-disclosure and perceived usefulness appearing to contribute to these improvements. The active and kind personality of the social chatbot, along with its capacity to provide empathy and comfort, seemed to have delivered a social support effect. To use social chatbots more effectively as a proactive intervention, it is necessary to address issues such as excessive anthropomorphism and inconsistent memories of personal details.

## Data Availability

Data supporting the findings of this study are available upon request from the corresponding authors. The data are not publicly available due to privacy concerns.

## Abbreviations

AI: artificial intelligence
LLM: large-scale language model
PHQ-9: Patient Health Questionnaire-9
ULS: UCLA Loneliness Scale
LSAS: Liebowitz Social Anxiety Scale
PAs: positive affect score
NAs: negative affect score
GAD-7: General Anxiety Disorder-7
PSS-10: Perceived Stress Scale-10
